# Seventh Annual Session of the Maryland State Dental Association

**Published:** 1890-03

**Authors:** 


### SEVENTH ANNUAL SESSION OF THE MARYLAND STATE DENTAL ASSOCIATION.

The annual session was held on December 5 and 6, 1889, at the St. James Hotel, Baltimore, the president, E. P. Keech, M.D., D.D.S., in the chair.

*Thursday, December 5, 1889.—Morning Session.*

PRESIDENT'S ADDRESS.

Gentlemen of the Maryland Odontological Society and the Maryland State Dental Association,—Before proceeding to the business which has called us together, I beg to trespass for a few moments upon your time and patience by giving expression to some thoughts which have suggested themselves to my mind as not inappropriate to the occasion. We have met to consolidate a union between two scientific bodies of this State,—the Odontological Society and the Maryland State Dental Association,—with a view of stimulating the members of each to renewed interest in the pursuit of the truths of science, and of adding force and strength to such pursuit by concentration and harmony of action. To the lay world we are only dentists, whose office it is to minister to the human teeth, or to supply their want by artificial appliances. This superficial and erroneous limitation of our professional entity is doubtless due to a defective nomenclature. So, too, the student who has burnt the midnight oil in acquiring a knowledge of the intricate truths of anatomy, physiology, and pathology is a mere surgeon,—chirurgeon,—handworker, because the visible demonstration of his knowledge is the skilful excision of a leg or a finger; the theologian, who in the pursuit of his divine science, has grasped the sublime relations existing between God, the Creator, and man, the creature, with all their obligations and responsibilities, degenerates into a mere preacher, whose principal office is to relieve the tedium of a Sabbath evening, or to lull into blissful repose the uncomfortable suggestions of a too sensitive conscience. After all, there is much in a name, even if a rose would smell as sweet if called a turnip. Our profession is a specialty; but this fact, while it certainly enhances its efficiency and usefulness, should not detract from its dignity. In our day, throughout the whole world, the tendency of all thought and action, both in science and art, is to specialties. So enormous is the multiplication of human knowledge that the average life of man is too short to even grasp, let alone master, it in all its essential details. Hence has arisen the necessity for a division of labor, mental as well as physical. The study of the telescope and the microscope alone, which, as has been well said, "enables the vision of man, far less acute than the eagle's, to range from the fixed stars to the twenty-thousandth of an inch bacteria," would compass an ordinary lifetime. In the mechanical and in the fine arts,—from the building of the Eiffel tower, the construction of the Brooklyn bridge, the breathing of almost life into the dead marble of a dying gladiator or a Venus of Milo, to the fashioning of a lady's slipper or the production of the pin and needle for domestic use,—all are the result of the skill and aptitude of the specialist. So in the science which has for its object the prevention or cure of those ills to which flesh is heir. The eye, the ear, the lungs, the heart, the nerves, the skin, every organ and function of the body, has its specialist. Why not the mouth, the most important of all, if any comparison be admissible between organs, each of which is necessary for the healthful and perfect operation of the whole? No one of these specialties is in itself a science. Oculism is no more a science than dentistry; dentistry no more than orthopedy. The proper understanding and practice of each do not depend upon its exclusive study,—as well expect to learn to swim in a bath-tub. The mental vision of the student must take a wider range. The would-be swimmer must strike out boldly into the wide lake or wider ocean. The vital phenomena of organized bodies, the composition and certain properties of material substances, the structure of organized bodies as learned from dissection,—physiology, pathology, chemistry, and anatomy,—these are the four great streams of science, separate and distinct, but together forming the one pure fountain from which every one who aspires to real eminence in any one specialty must drink deeply, or fail in his aspirations. We of the dental profession, gentlemen, have reason to congratulate ourselves upon the progress which, as a scientific calling, we have made. But yesterday we were sneered at as artisans,—hand-workers; to-day the curriculum of no respectable medical school is considered complete without a chair of dentistry. Perhaps no branch of the healing science has made such great progress and bestowed upon mankind such lasting benefits in so short a time as ours. The one discovery alone of the unfortunate Morton, the application of chloric ether to the operations of surgery, caused the great philosopher Lecky to assert that "the American inventor of the first anæsthetic has done more for the real happiness of mankind than all the moral philosophers from Socrates to Mill." True it is that we have not yet weeded out the ignorant charlatans, whose flaming advertisements and huge signs, hideous with grinning teeth and blood-curdling instruments of torture, still greet us in the thoroughfares. But let us hope that their day of darkness is short and will soon come to an end, to be succeeded by a race of practitioners to whom the steady beam of the pure rays of science will be more precious than the meretricious glitter of the vulgar shekel. The union which we are about to form can accomplish much in this direction. Science is not art, nor is art science. Yet each is necessary to the other, and should be inseparably blended. The former without the latter is a tree without fruit; the latter without the former is fruit, but dead-sea fruit, which turns to ashes on the lips. The practice of the healing art, in all its various specialties must mean, and in truth be, the application of the knowledge derived from the study of the four pure sciences which I have named, or it must be mere handicraft applied haphazard, without certain and known results. As tending directly to this much-to-be-desired consummation, it is gratifying to note the action of the National Association of Dental Faculties in the adoption of resolutions requiring the colleges to exact an examination preliminary to matriculation, and compelling students to attend a three years' graded course. This is an upward step in the right direction; and, in connection with two other great reforms,—the exclusion from the graduating class of mere practitioners of five years' standing, and the establishment of the graded course with preliminary and junior examinations, to both of which we are indebted to this same admirable association,—will at no distant day entitle the dental profession to an equal rank with any other body of scientific men, in dignity, in usefulness, and in education. The theme, gentlemen, as it unfolds itself before me is an inviting one, but I must kindly forbear and permit you to go on with your work. My purpose is to stimulate the thought, and to help the effort which is now happily reaching out everywhere for the higher and better education of our useful profession, in which, I am sure, I have the sympathy of every one of you, and which, I feel very certain, will be greatly promoted by the union which we are about to form.

Many thanks for your kind attention.

DISCUSSION OF THE PRESIDENT'S ADDRESS.

*Dr. A. J. Volck.*—Among the subjects presented by the president there is one which, I think, ought not to be allowed to pass without some special reference to it, and I desire to say a few words upon it. I refer to the allusion to the fact that, with but one exception, no dental society in the city of Baltimore or in the State of Maryland has ever succeeded.

Mr. President, you have handled that subject very tenderly, very daintily, and, I may say, with kid gloves. It is therefore right that somebody should handle it with the bare knuckles. The cause is not properly attributable to the fact to which you have referred. The cause lies in the difficulty between the two dental colleges in this city. And the sooner these colleges arrange the difficulty the better it will be for themselves and for the profession. Both are marshalled under the same banner; both are laboring to promote the same good purpose and the same great object; and I undertake to say here that the man who, as president of this association, will take this matter in hand, and will bring these two colleges together and have them join hands (and it may be your duty, Mr. President, to do it; and if so, may you succeed), that man will deserve the thanks of the profession of the whole State, and not only of the profession of this State, but of the profession at large. We have in this city the first dental college ever established in the world, and this fact should stimulate us to assist in any effort to remove the difficulty referred to. We are, as the president has well said, a body of men perfectly able to make a dental society successful, to undertake all the business and to perform all the duties of such an organization, but this same difficulty has constantly confronted us. If the members of the faculty, or the graduates of one college, are seen two or three times in a meeting of a State society, or in any other society, those of the other college uniformly stay away from such meetings. When we propose to elect an officer, we have to be very careful not to select a gentleman who has any connection with either one of the colleges, lest one of them should be given advantage over the other.

That is the cause, the only cause, why no dental society, with one exception, has succeeded here. I wish to call particular attention to that one exception. There is one dental society that has succeeded. It has not been in existence for more than a year and a half, but it meets monthly, and is a source of great benefit to its members. Its membership is made up of men who do not tolerate any jealousies of one another, who entertain the sincerest feelings of good fellowship, who come together for mutual improvement, and to have a good time. I make this statement because I do not want it to go upon record that no dental society in Baltimore has ever succeeded. The Association of Dental Surgeons of the city of Baltimore has been eminently successful, and it is a society in which there is not a speck of decay.

*Afternoon Session.*

H. B. Noble, D.D.S., Washington, D. C., read a paper on

### DENTAL HYGIENE.

Health being the corner-stone of all good work, either of body or mind, it should be the aim of every one to study and work for it. Our body is governed by fixed laws, which, if violated, will bring trouble just in proportion to the extent that the laws of hygiene are disregarded. The dentist, of all men, cannot afford to be careless of his health or of the laws of hygiene so nearly allied to it. A good dentist means a healthy, clean man; his personal habits must be those of a gentleman. Out-door exercise, in the pure air of heaven, should be regularly had by walking or riding.

In choosing an office, its hygienic surroundings should always be considered. Light and ventilation are two of the essentials. Light is of the first importance; north, south, and east, each has its advocates, and each has its advantages and disadvantages. From experience and observation we believe a southern exposure and south light the best; there are health-giving properties in every ray of the sun; it is the strongest and longest, and if properly regulated by white curtains in the middle of the day, you have an all-day light, and a friendly assistant, giving health and cheerfulness to all. In the middle of the day, in the summer months, when the direct rays of the sun are oppressive, before it reaches the south window it is so high it does not give you its direct rays. Our hottest weather is almost invariably accompanied with southern breezes, giving us good air as well as light.

The east is a good morning light, but is weakest at the closing hours of the day, just when one is tired and wants the best light possible. The north is a steady light, is not as changeable as a south light, and is claimed, by artists, to bring out the difference of shades of color with more distinctness than any other; but for our work, where strength is the element most needed, it is not equal to a southern light. A west light should never be chosen if either of the others can be had, as it is weak in the morning, and the direct rays of the afternoon sun must be shaded from a patient's face, making it unpleasant and weak when you get it.

Plenty of air and good ventilation should always be in order. The operating-room should be large and airy, and the best and brightest, not a little back corner, poor light, worse ventilation; privacy is all very well in its way, but not to the exclusion of light and good ventilation. Pure air and plenty of it is of prime importance to health; yet how little regard seems to be given to it, judging from the close medicinal odors that greet our olfactories in many of our dental operating-rooms, charged with the concentrated odors of creosote, carbolic acid, iodoform, and half a dozen other vile-smelling compounds, sweetened, perchance, with the odors of dead pulps, foul teeth, and sour stomachs. We get so accustomed to these vile, unhealthy odors that no effort is made to correct or remove them, requiring both patient and operator to exist, not live, in them day by day, until insulted nature rebels at this foul treatment, and enters her protest through headaches, backaches, weak eyes, and the last more fashionable trouble, "nervous prostration;" if he dies, they call it "*heart* failure," the last undoubtedly correct in all cases of death; but which gives not the slightest hint or indication of the primary cause of the disease.

Have all odorous medicines in glass-stoppered bottles, and in a closed case.

It is not necessary to smear your instruments with creosote and iodoform, and so inoculate your person and clothes as to advertise your calling. I have little doubt that disease has been communicated by foul, unclean nerve broaches, which always should be disinfected after using. No instrument that has been used should be put into another mouth until carefully cleansed.

The laboratory should, if possible, be a large, light, sunny room, with good draughts and ventilation; not the little back closet or dark cellar, so often seen; light and air must be had if good work is to be done.

There is no profession so likely to neglect exercise in the open air as the dentist, with a tired back to call and urge him to the lounge or easy-chair, when he should be filling his lungs with pure air by riding, driving, or walking.

The dentist has duties in hygiene outside his own health that should engage his attention; children and parents need advice, as well as professional work. How few persons, especially children, do we find who take proper care of their teeth? It should be our duty to instruct our patients in the duty of properly cleaning their teeth, and to give careful, minute instructions as to the manner of using the brush, so that there shall be no food for the bacteria to build nests for work on the pearls we are to watch and preserve. Instruct your patients that it is not the sweet things but the acid produced by fermentation in the mouth that decays their teeth.

Study the general health of your patient; it will often give you a clue to some hitherto misunderstood trouble. No part of our body can be diseased but it affects, more or less, every organ and tissue.

Nothing but a constant study of the laws of hygiene will enable us to ward off disease, especially that of the oral cavity, which we, as dentists, are called to look after and to treat; and as prevention of disease is better than cure, it becomes us to see that we are competent instructors in the laws of hygiene in its relation to the oral cavity.

We should educate our patients to greater care in the sick-room, that some antacid or mild germicide like listerine be used to rinse the mouth, as nearly all conditions of the sick are favorable to active bacterial growth, especially so in fevers, and many a bad decay dates its beginning from the sick-room, and we should improve every opportunity to urge our medical friends to greater care in this direction.

We should instruct children in the use of the tooth-brush, and oftentimes the parent needs the instruction and advice quite as much as the child.

Let us emphasize the mouth as a hygienic organ, whose functions, I fear, have never been properly appreciated by the medical profession, or overestimated by the dental. Health depends on good blood; unhealthy food, whether from inherent defect, bad cooking, or imperfect mastication, will not make good blood. The caterer of the stomach is the mouth, and disease there will tend to vitiate all the supplies of the stomach. How much of our food reaches the stomach through the portal of a diseased, and often a filthy mouth that is not in condition for proper mastication and insalivation in this active office of preparation, and thus is improperly prepared for passage into the alimentary canal.

Nature, in her prodigal care for the health of all her creatures, has placed at the portal of this great alimentary highway the teeth, which, for fitness for their functions, cannot be surpassed by any other organ of the body.

All healthy food must have its wedding-garment on, and this can be obtained only in the mouth, where a semicircular band of trained soldiers stand shoulder to shoulder to give welcome to the guests of the body.

Dr. J. Y. Crawford, in his address to the Southern Dental Association, at Galveston, last August, gave a vivid description of these guardians of health when he said,—

"The first effect is upon the nervous system. The grateful smell, as the food passes the lips, salutes the plexus of nerves that engrave with rich tracery the nasal arch and is conveyed to the sensorium. The lips open and the teeth seize the delicious morsel that comes thus heralded. Every nerve of taste tingles with gustatory pleasure, and summons, as with an electric bell, the liveried servitors who wait in this banqueting house of the gods. From lip to pharynx, floor, wall, and arch pour out their welcome. The nerves of taste carry joyous messages to the brain, and throughout the whole system glands—labial, buccal, palatine, molar, and lingual—open mouths of welcome on the free surface of the mucous membrane, and pour libations along the margin and dorsum of God's banqueting-table, the tongue. The *major domo*, the steward and the butler, the parotid, the submaxillary, and the sublingual glands send up from the cellarage salivary nectar through the ducts of Steno, Wharton, and Rivinus. The incisors carve, cuspids pierce, bicuspids tear, and molars grind around the whole royal arch from condyle to symphysis,—divine cutlery and plate, not of steel nor of silver nor gold, but of peerless enamel,—till at last the precious mouthful is delivered to the constrictor, stylo-, palato-, and salpingo-pharyngei to be safely conveyed through the œsophagus to the stomach, where it is taken up by the systemic economy and the digestible converted into chyle and blood to nourish the body, and the indigestible rejected and excreted."

This intricate machinery, so graphically described by our Southern brother, requires to be kept clean, if we would not poison the body, at every meal, by mingling with the food particles of decayed animal or vegetable matter.

Mingled with such carrion juices and micro-organisms, the most delicious food reaches the stomach in a condition destructive to health.

Scientific investigations have demonstrated that micro-organisms are potent disturbers of the normal conditions of the body, and are prime originators of many diseases, and the continual swallowing of these fungi in great numbers may produce serious disease.

Many of the diseases incident to childhood are now believed to be aggravated, if not induced, by these bacteria, and in many cases can be mitigated, if not prevented, by maintaining clean and healthy surroundings, especially that of the mouth.

Dental art and science is, we affirm, a most important branch of the healing art, and should be taught in all our schools, not in the cursory, indifferent manner natural to those who have not given it special study, but should be taught by those trained in dental schools, and who feel and know its importance.

The lack of proper care of the teeth in early youth often causes ill health and suffering, and this because the child, or parent, had not been properly instructed in the care, or importance to health, of good teeth properly articulated.

The dental surgeon who has preserved the teeth from decay has preserved the health, and rendered life more comfortable.

Let us as watchmen and guardians of the portal of this sacred temple see that nothing enters to deface any of the pillars that adorn the oral cavity, or that shall detract from their beauty; thus our patients' teeth will be like those described by the prophet Solomon,—

"His teeth will be like a flock of sheep that are all even shorn."

DISCUSSION OF DR. NOBLE'S PAPER ON "DENTAL HYGIENE."

*Dr. R. B. Winder.*—A valuable paper such as that which we have heard ought not to pass without due acknowledgment and the discussion to which it is entitled. We shall doubtless all agree as to the necessity for special education in this direction. There is nothing in the paper that can be harshly criticised, all its points being well taken, so far as I can see. The want of proper hygiene is very clearly indicated by the secretions of the mouth becoming vitiated, especially in low forms of fever. Under these circumstances decay in the teeth takes place rapidly. We all know now that caries of the teeth is caused by the presence of bacteria, and that these are generated in vitiated secretions. Since it has been demonstrated (and I consider that the fact has been clearly and absolutely demonstrated) that the health of the teeth depends on the chemistry of the mouth, it is obviously of the utmost importance to keep the mouth as clean as possible under all circumstances.

I regret to say that I am compelled to concede the truthfulness of all that has been said by the essayist in regard to what I may term the almost criminal neglect on the part of medical men *per se* in the matter of dental hygiene. This is so apparent that it might well be inferred that the teeth are ignored by them, or are regarded as undeserving of their attention. I do not say this in any unkind spirit, because I entertain the highest respect for the medical profession, but I mention it as showing that dentistry has not been incorporated in the curriculum of the medical student, and that the text-books practically ignore education in this direction. It is for this reason that dentistry is to-day a profession separate and distinct from that of medicine. The attempt was made in the early history of our profession to secure the co-operation of medical men in our work, but it proved ineffectual. I can give you no more striking illustration of the consequences which follow from this condition of things than that which was shown in a case in my own practice a few years ago. The case was that of a lady in feeble health, who applied to me to have her front teeth extracted. Her physical condition was not, in my judgment, such as to warrant the operation, and I suggested a preparatory method of treatment, to which she assented. I then inserted plastic fillings for temporary service until gold fillings could be put in. This was in the month of February. About the last of the following May she sent for me to come to her house, and, upon visiting her, I found that nearly all of the oxyphosphate fillings which I had inserted had been dissolved or washed out, this being due to an alkaline reaction from the saliva or secretions of the mouth. The decay had reached close to the pulp at certain points, and the sufferings of the patient were consequently quite severe. While it was apparent to me that the secretions were strongly alkaline, she informed me that she had been required, by her physician, to abstain from the use of acids because they would aggravate the indigestion from which she was suffering. I had said to her, in a humorous way, "You have been indulging in too many pickles, too much acid;" when, throwing up her hands in alarm, she assured me that her physician had strictly prohibited acids, although she had a most intense longing for them. Upon learning that her physician was a gentleman with whom I am well acquainted, one whom I knew to be a progressive and intelligent member of his profession, I took the liberty of preparing for her a lemonade, and advising her to make use of acids; in other words, I suggested an acid treatment. When her physician, in compliance with the request I made to her, called upon me, he assured me that my diagnosis of the case was absolutely correct, and, instead of objecting to what I had done, expressed his obligation to me for it. I had been fully confident, however, that in what I did I was not acting upon any uncertainty as to the kind of treatment the patient needed. I knew that oxyphosphate fillings would resist the action of all acids, but were easily dissolved by alkalies, and particularly by ammonia. I realized at the outset that the diagnosis of her physician was faulty, for the reason that, instead of making a test of the secretions of the mouth to ascertain whether they were alkaline or acid, he had arbitrarily assumed that her condition was acid. The result was that, after the patient had been placed on an acid treatment for a couple of months, her condition showed marked improvement; indeed, I have rarely seen more decided evidences of rapid recuperation than were shown in the case of that patient.

That was one instance corroborative of what Dr. Noble has said in regard to the condition of the mouth being absolutely overlooked by the attending physician, and the duty which is sometimes imposed upon us in forcing the matter upon the attention of the physician. In ill health the secretions of the glands become vitiated, and decay in the teeth results; and it often happens that when a patient whose teeth we have preserved in good condition returns to us, after a spell of sickness, we find that our work is a wreck, and that previous operations must be repeated, when a little care and attention on the part of the physician would probably have prevented this.

In the case of which I have spoken the use of litmus paper would have enabled the physician accurately to diagnosticate the condition of the mouth. This paper is blue and red. The blue, when applied to an acid, turns red; and the red, upon touching anything that is even slightly alkaline, turns blue, thereby furnishing a very delicate means of testing the mouth. If tested by this means, the physician would have found that the condition of the mouth was alkaline, not acid, and he would have prescribed acids instead of restraining the patient from the use of them. The symptoms, in sickness, which are indicated by a furrowed tongue and a constant bad taste in the mouth, are well known; and if the physicians would devote the requisite attention to these and kindred symptoms, their treatment would prove more effectual and salutary.

In regard to what has been said in the essay about the arrangement of an operating-room, with reference to light and in other respects, I desire to say a word, as the subject is one of general interest to the profession. I have never yet seen an operating-room the arrangement of which was, in my opinion, based on scientific principles. I agree with Dr. Noble in all he has said concerning the necessity for ventilation, the ingress and egress of pure air, and the avoidance of all disagreeable odors. Some practitioners would have in the office all the light that they could possibly get. I suggest that what we want are direct rays of light, and that we do not need to have a reflecting surface from which those rays may be returned or reflected back as from a looking-glass. I think that which is most desirable is an arrangement similar to a photographer's, with a background, and so arranged that, when they have passed beyond your head-rest, the rays may stop and be absorbed. I repeat that the best arrangement seems to be one by which you have a direct light upon an objective point, without any returning or interfering rays.

With regard to the color to be used in the papering of the walls of an operating-room or office, I suggest that if our preference is to be determined by the fact that nature has clothed the earth in a certain color, we should of course select green. An additional consideration in favor of this color is to be found in the generally-admitted fact of the beneficial effect of green in resting the eyes. The shades usually worn over the eyes of students are of a green color. I can say, from my own experience, that, having at one time had a laboratory commanding a view of surrounding foliage, I often experienced a sense of relief, when engaged on critical work, in looking out upon the green fields and trees within range of my vision. There is, however, a consideration that may be urged as an objection to this color, and that is, that green-colored wall-papers, as I have learned from manufacturers, are made by an arsenical preparation, and therefore may contain poisonous substances which would have a deleterious effect upon the atmosphere of a room or in some other way prove prejudicial to health.

*Dr. Wm. A. Mills.*—Mr. President, I was glad to hear Dr. Noble call attention to the character of some of the colored papers on the walls of our offices, and particularly in the rooms in which we operate, and in our sleeping-apartments. I regard this as one of the greatest sources of danger to which members of our profession could expose their lives. I know of an instance in which a family was poisoned (though the cause of the trouble could not be ascertained at the time), where it was afterwards discovered that the curtains or hangings in their apartments had been prepared with arsenical compounds of some kind. I think that in advocating hygienic laws, and seeking to make a practical application of them in the arrangement of our dwellings and offices, we ought to be very careful in the selection of paper-hangings, and that we ought never to prefer green as a color.

*Dr. A. J. Volck.*—Mr. President, why not follow the rule which is followed by the artist in a matter like this? The artist wants a concentrated light upon his easel just as we want it upon our patients. He papers his operating-room with colors which are absorbents of light. Therefore he prefers, generally, a maroon or some dark-gray or broken color such as you find in gray wrapping-paper. I know of one studio in New York which is covered with hemp cloth, which material not only makes a very good appearance, but an exceedingly pleasant appearance to the eye.

Green is a very good color when looked at in the open air; the effect presented by green woods or green fields is very pleasant; but a room, the covering of which is wholly of green, is one that affords no relief to the eye, and is anything but a relief to it, because the green reflects the light more than does maroon, gray, or other colors. It does not absorb the light as those colors do. Green is a secondary color, while the others are tertiary. Being less positive, the other colors are more pleasant to the eye.

*Dr. Charles D. Cook.*—Mr. President, having come in late and heard only a portion of the paper, I do not think I can add anything to what has already been said. If I may be allowed, I will make one suggestion, and that is, it is customary for dentists to speak of dentistry as though it were a profession separate from and independent of the medical profession; those who hold the degrees of M.D. and D.D.S. are in the habit of speaking of it as a profession; whereas they should recognize dentistry as a specialty. I think we ought to treat the general practitioner and the dentist alike as medical men; that the general practitioner should be known as such in contradistinction from the dental specialist; and that we ought not to assume that our art or our practice is fundamentally different from that of the general practitioner.

In our code of ethics we almost invariably start out with the proposition that dentistry is a specialty of medicine, and that we are medical men. The tendency now, among teachers and among the more advanced of our brethren, is to train dental students not only as specialists but as medical men. In our colleges such students are, I think, being trained from a medical stand-point, as they have been in England for the last twenty-five years. In London, the dental student receives the same training in anatomy, physiology, therapeutics, etc., that the medical student receives; the teachers being the same for each. At the examination in the Royal College of Surgeons, where the degree of L.D.S. is conferred, there are two special examiners on the specialty of dentistry, who are also members of the Examining Board of the Royal College of Surgeons. The only difference between the two classes is that the dental students receive a special training for their work, in the filling of teeth, in making obturators, etc., and have a special school just as the oculists have. So that, practically, all the graduates are medical men, but the dental students receive this special degree. I think we owe it to ourselves to speak of our practice as a specialty, and to drop the term "profession" except so far as it applies to the general profession of medicine.

*Dr. H. B. Noble.*—Mr. President, I have but a few words to say. One of the positions which I have taken has been freely criticised, but all the speakers have agreed upon the main point. In regard to the papering of our rooms, much that has been said may be found to be of value. I think that the question as to the most advisable color to be made use of should receive more attention than it has yet received. I am not prepared to say what color I regard as the best, but undoubtedly there are certain colors which are preferable to others. I think that the subject has been sufficiently ventilated to lead the members to consider it thoroughly, and that the result of their deliberations may eventually enable us to get our offices in as perfect order and condition as is possible.

Subject passed.

